# The Effect of Combined Drought and Temperature Stress on the Physiological Status of Calcareous Grassland Species as Potential Candidates for Urban Green Infrastructure

**DOI:** 10.3390/plants12102003

**Published:** 2023-05-16

**Authors:** Jacek Krzyżak, Szymon Rusinowski, Krzysztof Sitko, Alicja Szada-Borzyszkowska, Jacek Borgulat, Radosław Stec, Hans Martin Hanslin, Marta Pogrzeba

**Affiliations:** 1 Institute for Ecology of Industrial Areas, 6 Kossutha Street, 40-844 Katowice, Poland; j.krzyzak@ietu.pl (J.K.); krzysztof.sitko@us.edu.pl (K.S.); a.szada-borzyszkowska@ietu.pl (A.S.-B.); j.borgulat@ietu.pl (J.B.); r.stec@ietu.pl (R.S.); 2 CommLED Solution Sp. z.o.o., 149 Tarnogórska Street, 44-100 Gliwice, Poland; szymon.rusinowski@commled.eu; 3 Plant Ecophysiology Team, University of Silesia in Katowice, 28 Jagiellońska Street, 40-032 Katowice, Poland; 4 Division of Environment and Natural Resources, Norwegian Institute of Bioeconomy Research, P.O. Box 115, NO-1431 Ås, Norway; hans.martin.hanslin@nibio.no

**Keywords:** nature-based solutions, drought, heat, calcareous grassland species

## Abstract

Nature-based solutions are promising for climate adaptation and environmental management in urban areas, but urban conditions are stressful for vegetation. In particular, the interaction of drought and high temperatures may be detrimental. Guiding plant selection for urban greening with native species requires a far better knowledge of plant adaptations and stress acclimation. We tested the physiological responses of four candidate calcareous grassland species for green roofs and walls to the combined effects of drought and high temperatures under controlled conditions. The tested species proved relatively resistant to stress despite different strategies to protect the photosynthetic apparatus, maintain water balance, and repair damages. Based on the physiological responses, we rank the species in descending order of resistance to the stress factors tested: *Trifolium medium* > *Festuca ovina* > *Carex flacca* > *Potentilla reptans*, but all four can serve as potential candidates for green walls and roofs. Physiological stress screening of plant species for use on green roofs and walls supplements the habitat template approach to provide a stronger and wider base for prioritizations.

## 1. Introduction

Urban nature-based systems such as green roofs and walls, rain gardens, bioswales, and parks have the potential to support stormwater management, reduce the urban heat island effect, and promote biodiversity and esthetic qualities [1,2,3]. However, such uses of nature-based solutions to support urban climate change adaptation place high demands on vegetation’s adaptive capacity and ability to acclimatize to fluctuating and combined stressors. Heat, air and soil pollution, fluctuating soil moisture, and limited rooting volumes combine to create complex urban stresses with negative impacts on vegetation [4]. In particular, extreme weather events during the growing season, such as the combination of drought and high temperatures, can have a detrimental effect [5]. This requires species that are resilient enough to withstand stressful conditions over multiple growing seasons, especially for the limited soil volumes and harsh environmental conditions of green roofs and walls [6,7], and all this needs to be in a low-input, low-maintenance environment where urban greening contributes to carbon-efficient and sustainable urban solutions [8].

To overcome these limitations and provide ecosystem services, plant selection should meet several requirements [9]. The selected plants should have minimal fertilizer and water requirements and contribute to native urban biodiversity. Species must also have physiological tolerance and acclimation to fluctuating combined stressors and be flexible at maintaining water status and photosystem function. As green roofs and walls are used for stormwater management, plants should have flexible water use, with high water use in moist soil, but also be able to maintain turgor under dry conditions [10]. Progressive water deficit manifests itself at the cellular level to the whole plant, with rapid changes in the transcriptome, metabolites, assimilation, and respiration, followed by reduced growth and morphological adjustments [11]. Both drought and temperature stress adversely affect plant growth, development, and physiological status [12]. Chlorophyll a fluorescence is a well-known and widely used method of evaluating the performance of the photosynthetic apparatus [13]. Pigment content and oxidative stress enzyme activity are also sensitive plant markers of abiotic stress [14]. Monocotyledonous species are better adapted to drought compared to dicotyledonous species, especially due to them maintaining water status during water deficit [10,15]. Therefore, grasses are good candidates for roofs and walls, but their contribution to biodiversity is limited.

Species for green roofs are often selected using a habitat template approach [16], where species are selected from natural systems with shallow soil and comparable abiotic conditions. In Central Europe, calcareous grasslands could provide a suitable plant genetic pool for this task. Calcareous grassland species are well adapted to drought and heat of the Central European landscape, and therefore can likely benefit from climate warming by increasing the potential habitat area [17]. More specifically, this type of grassland provides valuable habitats for many specialized, rare, and endangered plant or insect species, and is therefore considered a key area for biodiversity conservation in agricultural landscapes [18]. Nutrient availability is low [19] because the decomposition rate is low [20]. Low nutrient availability can stress organisms by limiting resources, while calcium carbonate itself can contribute to additional phosphorus immobilization [21]. Therefore, both monocot and dicot species exist in this system, but knowledge of their responses to more extreme combinations of water deficit and high temperatures as part of their adaptations is limited.

Although appropriate vegetation and materials for green roofs and walls can reduce the need for irrigation, the frequency of severe droughts and heat waves requires a backup system to ensure vegetation survival [22]. Such systems can be automated using sensors, irrigation systems, and/or fertilizers, which increases the cost of planting but significantly reduces the labor required for installation [23,24]. Considering this, the most reasonable solution would be a combination of both approaches, which could significantly extend the life of the plant without external maintenance, with possible optimization based on the data collected by sensor and management systems.

To better understand the physiological adaptations and acclimation potential of species considered for green roofs and walls, we examined the effects of combined drought and temperature stress on the physiological status of a number of calcareous grassland species that are both monocotyledonous and dicotyledonous, and thus differ in growth form and phylogeny. We hypothesize that monocotyledonous species are more resistant to combined drought and heat stress than dicotyledonous species because of their ability to maintain turgor under water deficit/drought.

## 2. Results

### 2.1. The Effect of Drought and Temperature Stress on Growth, Gas Exchange, and Pigment Content

Simulated drought and heat did not cause any significant decrease in biomass or protein content in the shoots of the investigated plant species, with the exception of *Trifolium medium* (Tm) (Table 1). In *Festuca ovina* (Fo), an increase in biomass was measured in the drier variants compared to the control. On the other hand, a significant decrease in the content of chlorophylls and carotenoids was observed in all species under the influence of heat stress. The decrease in the content of plant pigments was accompanied by a significant decrease in the values of gas exchange parameters, such as transpiration and CO_2_ assimilation. At the same time, Fo and Tm showed an increase in water use efficiency in the drought variants compared to the control (Table 1).

### 2.2. The Impact of Drought and Temperature Stress on Photosynthetic Apparatus

**Figure 1:**
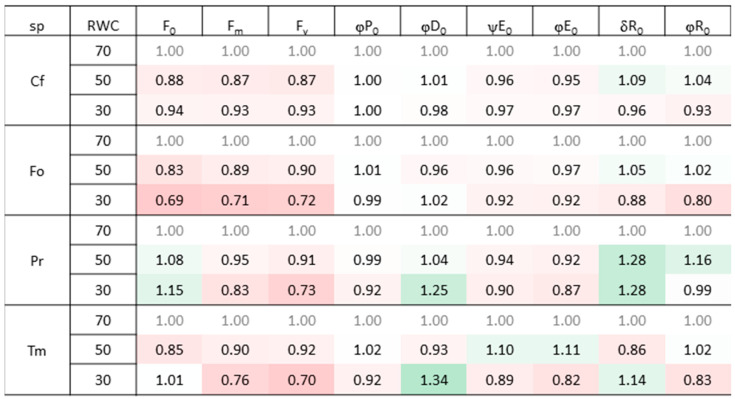
The effect of combined drought and heat on selected parameters describing the light-dependent phase of photosynthesis in investigated plant species. The results are presented as a heatmap, for which mean values were used (*n* = 5). The red coloring of the cell shows a negative correlation, while the green one shows a positive one. The intensity of the color informs about the strength of the correlation. Abbreviations: Cf—*Carex flacca*, Fo—*Festuca ovina*, Pr—*Potentilla reptans*, Tm—*Trifolium medium*, RWC—relative water content, F_0_—minimal fluorescence, Fm—maximal fluorescence, Fv—variable fluorescence, φP_0_—maximum quantum yield of primary photochemical reactions, φD_0_—quantum efficiency of energy dissipation, ψE_0_—values of probability that a trapped exciton moves electron into the electron transport chain beyond Q_A_^−^, φE_0_—quantum efficiency of electron transfer from Q_A_^–^ to electron transport chain beyond Q_A_^−^, δR_0_—probability that an electron is transferred to reduce end electron acceptors at the PSI acceptor side, φR_0_—quantum yield for the reduction of terminal electron acceptors on the acceptor side of PSI.

The strongest negative impact of drought on the efficiency of photosystems was measured in Fo, where the significant decrease in chlorophyll fluorescence parameters was observed compared to the control (Figure 1). At the same time, in both tested angiosperm species, *Potentilla reptans* (Pr) and Tm, a significant increase in dissipated energy (φDo) was measured with increasing drought. Drought and heat had the least impact on the *Carex flacca* (Cf) photosynthetic apparatus among the tested species (Figure 1).

**Figure 2:**
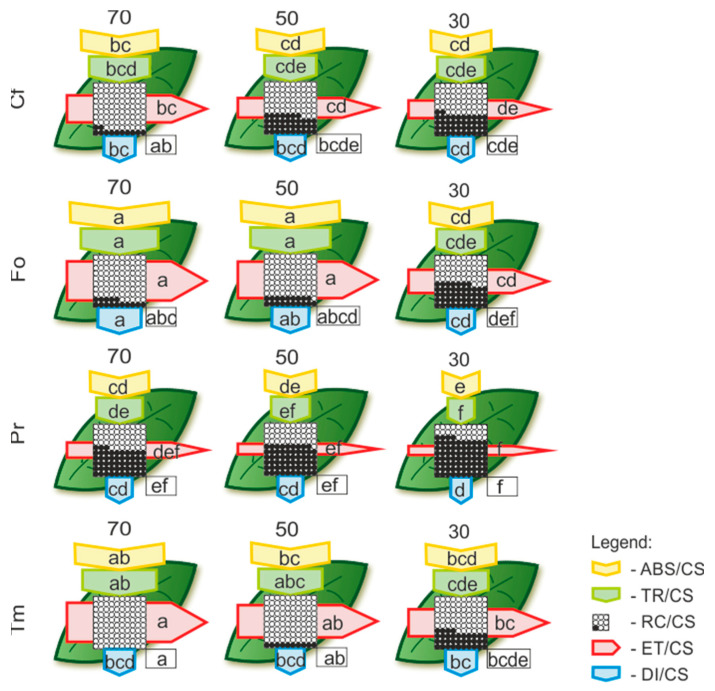
Leaf model showing the phenomenological energy fluxes per the excited cross sections (CS) of the leaves of selected plant species at the end of drought experiment. Each relative value of the measured parameters is the mean (*n* = 5), and the width of each arrow corresponds to the intensity of the flux. Yellow arrow—ABS/CS, absorption flux per CS approximated; green arrow—TR/CS, trapped energy flux per CS; red arrow—ET/CS, electron transport flux per CS; blue arrow—DI/CS, dissipated energy flux per CS; circles inscribed in squares—RC/CS, % of active/inactive reaction centers. White circles inscribed in squares represent reduced Q_A_ reaction centers (active), black (or orange) circles represent nonreduced Q_A_ reaction centers (inactive), 100% of the active reaction centers responded with the highest mean value observed in the control conditions. Means followed by the same letter for each parameter are not significantly different from each other using the Fisher LSD test (*p* < 0.05). Letters are inscribed into arrows, except for RC/CS, where they are placed in a box in the bottom right corner of the square with circles.

Models of energy flow through the photosystem II (PSII) of excited cross sections (CS) of leaves enable a quantitative and qualitative comparison of the efficiency of the light phase of photosynthesis between the studied species and their response to drought and heat stress (Figure 2). Under the control conditions, the Tm and Fo photosystems showed similar efficiency, while the Pr showed the worst. The analysis using the model confirmed a lack of significant differences in most parameters of Cf under the influence of drought. At the same time, it was shown that the best quantum yield in the conditions of the strongest drought compared to the other species tested was characterized by PSII in Tm (Figure 2).

### 2.3. The Effect of Drought and Temperature Stress on Enzyme Activity and MDA Content

**Figure 3:**
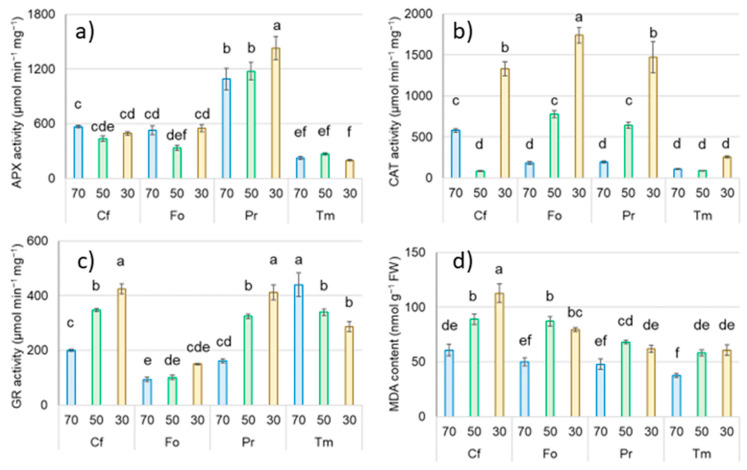
The effect of combined drought and heat on activity of APX (**a**), CAT (**b**), and GR (**c**), and content of MDA (**d**) in leaves in investigated species of sedge (Cf—*Carex flacca*), grass (Fo—*Festuca ovina*), rosaceous (Pr—*Potentilla reptans*), and clover (Tm—*Trifolium medium*). Data are means ± SE (*n* = 3). Means followed by the same letter for each parameter are not significantly different from each other using the Fisher LSD test (*p* < 0.05). Abbreviations: APX—ascorbate peroxidase activity; CAT—catalase activity; GR—glutathione reductase activity; MDA—malondialdehyde content.

The level of oxidative stress and associated enzymatic activity in response to drought and heat were definitely species-specific (Figure 3). Sedge under dry conditions did not show an increase in APX activity, with a significant increase in CAT and GR activity and the highest MDA concentrations among all the tested species under the influence of the tested stresses. Qualitatively similar responses to drought were noted for Fo and Pr, although the highest activity of APX and GR was measured in Pr, and CAT activity was highest in Fo, in response to drought. In the case of clover, there was no significant increase in APX and CAT activity under the influence of increasing drought, with a simultaneous decrease in GR activity; however, the effect of drought was a significant increase in the content of MDA in Tm leaves at the end of the experiment compared to the control (Figure 3).

### 2.4. Principal Component Analysis and Corelations

**Figure 4:**
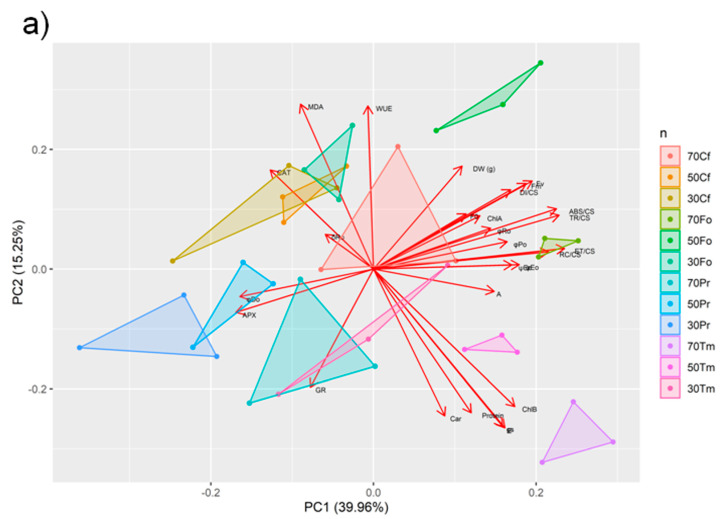

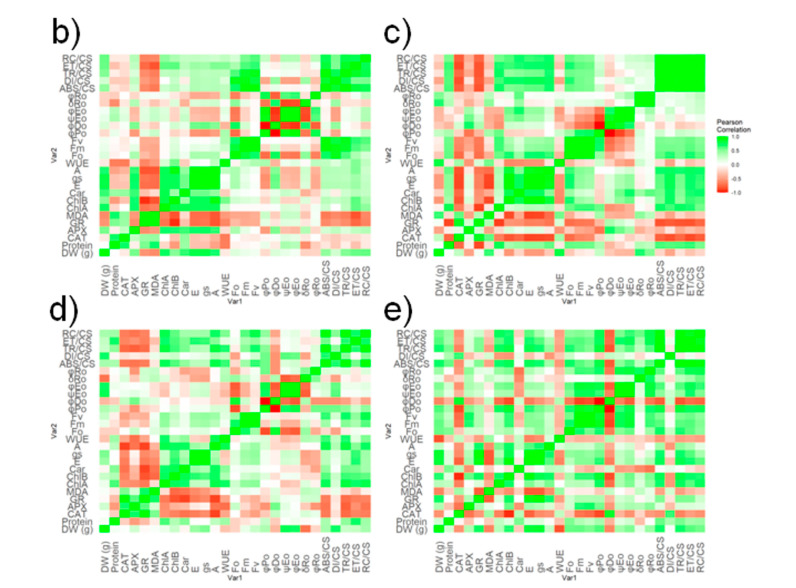
Principal component analysis (**a**) and heatmaps of correlation between selected parameters for *Carex flacca* (**b**), *Festuca ovina* (**c**), *Potentilla reptans* (**d**), and *Trifolium medium* (**e**) under combined drought and heat treatment. Abbreviations: DW (g)—dry weight in grams; Protein—protein content; CAT—catalase; APX—ascorbate peroxidase; GB—glycinebetaine; MDA—maliondialdehyde; ChlA—chloropyll A; ChlB—chlorophyll B; Car—carotenoid; E—transpiration rate; gs—stomatal conductance; A—photosynthesis rate; WUE—water use efficiency; F_0_—minimal fluorescence; Fm—maximal fluorescence; Fv—variable fluorescence; φP_0_—maximum quantum yield of primary photochemical reactions; φD_0_—quantum efficiency of energy dissipation; ψE_0_—values of probability that a trapped exciton moves an electron into the electron transport chain beyond Q_A_^−^; φE_0_—quantum efficiency of electron transfer from Q_A_^–^ to electron transport chain beyond Q_A_^−^; δR_0_—probability that an electron is transferred to reduce end electron acceptors at the PSI acceptor side; φR_0_—quantum yield for the reduction of terminal electron acceptors on the acceptor side of PSI; ABS/CS—absorption flux per CS (leaf cross section), approximated; DI/CS—dissipated energy flux per CS; TR/CS—trapped energy flux per CS; ET/CS—electron transport flux per CS; RC/CS—number of active reaction centers.

The PCA analysis confirmed that the tested species were characterized by different mechanisms of response to drought stress, because they formed separate groups arranged in a clear gradient consistent with the stress intensity (Figure 4a). Moreover, the correlations between the examined physiological parameters revealed species-specific patterns of response to drought and heat stress (Figure 4b–e).

## 3. Discussion

All species studied were affected by the drought and temperature treatments, and in most cases, the symptoms were more pronounced as the water deficit increased. Nevertheless, the response to the combined drought and heat stress was very species-specific, although our hypothesis of better adaptation to the combined stress was not supported in the monocotyledonous species [25]. Rayner et al. [26] reported that in a hot climate environment where summers are hot and dry, only plants with leaf succulence survived after 42 weeks of cultivation on the green roof without irrigation. Given extreme weather events, one might assume that alien species could be the solution. However, when creating plant communities for green infrastructure, the risks of biological plant invasions should be taken into account, including the selection of initial planting, maintaining a community that is not dominated by invaders, and preventing the spread of invasive plants [27]. In light of the above, it is important to note that urban ecosystems in general are not only entry points for many non-native species, but can also become focal points for dispersal into surrounding landscapes [28]. Consideration of potential risks in habitat design, together with conservation of local biodiversity, could be the solution. Species that could fit green infrastructure habitat templates are often found in rocky habitats, dunes, or other open areas where harsh conditions prevent forest formation [16].

In this work, all plants were possessed according to the habitat template, but they belonged to different functional groups. A comprehensive study of the physiological response of plants to drought and high temperatures is important for plant selection and/or irrigation schedules. It is not easy to directly determine which plant species is most sensitive to a combined drought and heat treatment based on individual parameters.

*Carex flacca* (Cf) was characterized by a significant decline in photosynthetic parameters, even when drought was not the least limiting factor. There was a huge decrease in gas exchange parameters, especially when comparing the control and the 30% RWC treatment. In drought conditions, the plant produces ROS, while as a response, antioxidants, flavonoids and secondary metabolites play a protective role in detoxifying the plant ROS and protect the plant against stress conditions by stabilizing the proteins and amino acids [29]. It seems that the combination of heat and drought stress increases the activity of glutathione reductase (GR) in the plant, while ascorbate peroxidase (APX) is not involved at all in the mitigation of stress. Volk et al. [30] reported for Cf that even small amounts of water in the soil can increase biomass production and gas exchange parameters. There is no further information in the literature on the response of this species to drought and temperature, but Gretter et al. [31] listed this plant as a potential species for extensive green roofs.

*Festuca ovina* (Fo) showed an interesting pattern, mainly associated with significant damage to the photosynthetic apparatus, which was mainly visible in the phenomenological energy fluxes of the leaves in the pipeline models. It seems that this plant could not maintain the efficiency of the photosynthetic apparatus at the same level under higher water deficit. Despite this fact, the reduction in efficiency at the lowest RWC was not visible in biomass production, as it remained unchanged compared to the control. In contrast to Cf, catalase (CAT) was the leading enzyme in the antioxidant apparatus, next to GR, which was much lower. Interestingly, the MDA content in Fo was lower regardless of the water treatment, especially considering the similar values in the control. Wang et al. [32] reported for different fescue species that *F. ovina* is characterized by high drought and heat tolerance regardless of the genotypic variation studied. Moreover, Khoshkholghsima and Rohollahi [33], comparing *F. ovina* with other grass species in Iran under drought stress, found a significant reduction in biomass only at higher water deficits. Using *F. ovina* on extensive rooftops, Vahdati et al. [34] noted that *F. ovina* might have problems establishing itself due to spring and autumn cold, which in turn could lead to midseason drought problems.

For *Potentila reptans* (Pr), it was found that drought treatments did not significantly affect its biomass production. There were visible symptoms in photosynthetic apparatus activity and efficiency; however, those are minor and related to the highest water deficit, particularly visible in gas exchange parameters. Considering antioxidative enzyme activities, all of those investigated were increased (APX, GR, CAT), while malone di-aldehyde (MDA) content was the lowest, and at the same level as for *Trifolium medium* (Tm) compared to the rest of the plants studied. There is not much information about this species in terms of physiology and cooperation with drought and/or heat stress, but it was directly suggested as a green roof species by Seyedabadi [35]. They reported that although *Potentilla reptans* (Pr) has excellent cold and drought resistance, its CO_2_ uptake performance is poor, which is also true for this study compared to other species studied. This makes it an unfavorable plant for use cases where the reduction of CO_2_ emissions is the main objective.

*Trifolium medium* (and other *Trifolium* species) are probably the most commonly used plant for green roofs in European climates [36,37]. *T. medium* was characterized by a significant decrease in photosynthetic parameters, but only at the highest water deficit, which was also true for the biomass produced by these plants. There is an interesting pattern for the antioxidant mechanism based mainly on the contribution of CAT; however, these values are much lower compared to other species studied, while APX reaches the lowest value among all plants studied, even for the control. The lowest value of antioxidant enzyme activity could be explained by the data of Li et al. [38], who showed that in *Trifolum repens* during prolonged drought, antioxidant enzyme activities increase in the first days of drought, then decrease and remain unchanged during the recovery period. Reynolds-Henne et al. [39] showed that the stomatal response of *Trifolium* to heat and drought is complex: legumes respond to moderate stress by closing stomata or irregular conductance and to high temperature stress by increasing stomatal conductance, even under drought. While the response to moderate overall stress indicates a tendency to favor water balance, the response to higher stress suggests that the metabolic effects involving protection of the photosynthetic apparatus from heat are limited. This observation was also confirmed in this work by the increase in water use efficiency (WUE), with a simultaneous decrease in stomatal conductance and transpiration. All the plants studied showed different mechanisms of coping with drought and heat stress, as well as different levels of resistance.

## 4. Materials and Methods

### 4.1. Experiment Design

The soil substrate for the pot experiment consisted of 70% sand (*w*/*w*), 5% garden compost (*w*/*w*), and 25% soil (*w*/*w*), collected from the calcareous grassland near Bytom, Poland. The collected soil was air-dried and passed through a 4 mm sieve to remove stones and plant debris. The soil substrate was manually mixed. The physicochemical characteristics of soil substrate are presented in Table 2. Pots with volumes of 2.5 L were filled with 3 kg of the substrate and planted with *Festuca ovina* L. (narrow-leaved grass), *Potentilla reptans* L. (forb), *Trifolium medium* L. (legume), and *Carex flacca* Schreb. (sedge). In total, 48 pots were planted (4 species × 3 treatments × 4 repetitions). In this paper, drought was defined as a decrease in relative water content from 70% in the control to 50% and 30% in experimental drought treatments. Plant material was collected during the field visit to the Upper Silesia and Cracow Jura calcareous grasslands (17 sites) and propagated from wild-collected material in the nursery established in the Institute for Ecology of Industrial Areas backyard. After plant acclimation during one growing season, seedlings were selected and transfer to the pot experiment. Plant size was uniform within species. Plants were grown in a phytotron under controlled conditions: temperature 22/16 °C (day/night), light intensity PAR = 300 μmol (photons) m^−2^ s^−1^, photoperiod 16/8 h, and relative humidity around 40%.

Pots were randomized on one of three levels of soil moisture control with relative water content (RWC) of 70%, 50%, 30%, with four replicates per species and treatment. During the first 6-week growth period, a relative water content (RWC) of 70% was applied in all pots. In the next phase of the experiment, irrigation was differentiated to achieve 70%, 50%, and 30% of RWC. Soil moisture was monitored daily with a WET sensor (Delta-T Devices, UK). Data from the device (volumetric water content) were calculated to RWC using calibration curve obtained for the tested soil substrate. The amount of irrigation water provided daily was based on the empirically measured full water capacity and the daily RWC measurement (Figure S1). After cultivating the plants under differentiated conditions for three weeks, an additional stress factor was applied to all treatments, including control, by increasing the air temperature to 30 °C for three days, which was defined as high temperature (heat) stress. Subsequently, the photosynthetic rate, intercellular CO_2_, transpiration, stomatal conductance, chlorophyll a fluorescence, and pigment content of the plants were measured. At day 3 of heat treatment, samples for enzymatic activities were collected and plant shoot biomass was determined.

### 4.2. Soil Substrate Physicochemical Parameters

The pH of the soil substrate was measured in H_2_O (ratio 1:2.5 *m*/*v*) and KCl using a combined glass/calomel electrode (OSH 10-10, METRON, Gliwice, Poland) and a pH-meter (CPC-551, Elmetron, Gliwice, Poland) at 20 °C. Electrical conductivity was determined with an ESP 2ZM electrode (EUROSENSOR, Gliwice, Poland) according to the Polish standard [40]. Soil texture was determined using the hydrometric method according to the Polish standard [41]. The total nitrogen concentration in the soil was determined using the dry combustion method [42]. The concentrations of available phosphorus and available potassium were determined according to the method described by Egnér et al. [43]. Total nitrogen (N) concentration in plants was measured by the titration method [44], while total phosphorus (P), potassium (K), calcium (Ca), and magnesium (Mg) concentrations in plants were determined by ICP (Liberty 220, Varian, Palo Alto, CA, USA) in previously mineralized samples (microwave sample digestion, ETHOS 1, Milestone, Sorisole, Italy) according to the procedure specified by the manufacturer (concentrated HNO_3_ and H_2_O_2_, 4:1 *v*/*v*). The sulfur content (S) was determined according to the method [45].

### 4.3. Plant In Vivo Measurements

Gas exchange measurements were performed using an infrared gas analyzer (LCpro SD, ADC Bioscientific, Hoddesdon, UK). Saturated photosynthetic rate (A), transpiration rate (E), and stomatal conductance (gs) were measured as previously described by Rusinowski et al. [46]. The LCpro SD was equipped with a narrow chamber (580 mm^2^) set at 22 °C, 1500 μmol m^−2^ s^−1^ for photosynthetically active radiation (PAR), and a CO_2_ concentration of about 400 ppm. Chlorophyll a fluorescence was measured using Handy Plant Efficiency Analyser (Hansatech Instruments Ltd., Norfolk, UK). Before analysis, leaves were dark-adapted for 25 min using specially designed clips (LC, Hansatech Instruments, Ltd., Norfolk, UK). Subsequently, a saturated pulse of 3500 μmol m^−2^ s^−1^ was applied for 1s to obtain readings. Five measurements were performed for single treatment on fully developed leaves.

### 4.4. Plant Enzymatic Activity

The activities of catalase (CAT), glutathione reductase (GR), and ascorbate peroxidase (APX) were determined according to the method described in Noctor et al. [47]. The enzyme activity was expressed as micro- (CAT) and nanomoles (APX and GR) per milligram of protein analyzed in the plant extract per minute. The protein content of the desalted plant extracts was determined according to the Bradford [48] method.

The content of chlorophyll a and b was determined colorimetrically [49], as was the content of carotenoids [50] using DMSO as the extraction agent. The amount of malondialdehyde (MDA) in the plants was determined spectrophotometrically according to the method of Hodges et al. [51]. Here, as well as in the case of plant pigments, the concentration in the homogenate was calculated to one gram fresh weight. Three measurements were performed for single treatment on leaf samples.

### 4.5. Data Analysis

The statistically significant differences among mean values were determined using one-way ANOVA and post hoc Fisher LSD test (*p* < 0.05). The statistical analysis was performed using the computer software Statistica v.13.1 (Dell Inc., Round Rock, TX, USA) and R v.4.2.0. (R Foundation for Statistical Computing, Vienna, Austria). The pipeline model of energy fluxes through leaves’ cross sections was made using CorelDRAW X6 (Corel Corp., Ottawa, ON, Canada). PCA was used to identify the dominant groups of the factors that determine and describe the variety differences. Relative coordinate spatial distribution maps of assimilable elements were constructed based on plot elemental analysis data and inverse distance weighted (IDW) interpolation.

## 5. Conclusions

The results obtained did not allow for a clear verification of the research hypothesis, which assumed a higher resistance of monocotyledonous species compared to dicotyledonous ones to combined drought and high temperature stress. The selected plant species are well—but variably—adapted to the environmental stresses of urban environments such as drought and high temperatures. *Trifolium medium* and *Festuca ovina* were found to be the most resistant species to drought and high temperature stress, based on principal component 1 (PC1), which identifies these species as the most photosynthetically efficient and productive in terms of biomass (right side of PCA coordinate system). In contrast, the species that responded worst to stress were *Potentilla reptans* and *Carex flacca*. Interestingly, monocotyledons and dicotyledons differ in oxidative stress parameters (bottom versus top of the PCA coordinate system). Nevertheless, all tested species proved to be relatively resistant to stress despite different strategies, which predestines them as species that can be used in nature-based solutions such as green walls and roofs.

## Figures and Tables

**Table 1:** The effect of combined drought and heat on growth, pigment concentration, and gas exchange of investigated plant species. Data are means ± SE (*n* = 3 for dry weight, protein, and pigments; *n* = 5 for gas exchange parameters). Means followed by the same letter for each parameter are not significantly different from each other using the Fisher LSD test (*p* < 0.05). Abbreviations: Cf—*Carex flacca*, Fo—*Festuca ovina*, Pr—*Potentilla reptans*, Tm—*Trifolium medium*.

sp	RWC	Shoot Dry Weight (g)	Protein Content (mg g^−1^ FW)	Chl A Concentration (mg g^−1^ FW)	Chl B Concentration (mg g^−1^ FW)	Carotenoids Concentration (mg g^−1^ FW)	Transpiration (mmol m^−2^ s^−1^)	Stomatal Conductance (mmol m^−2^ s^−1^)	Photosynthesis Rate (µmol CO_2_ m^−2^ s^−1^)	Water Use Efficiency
Cf	70	4.99 ± 1.34 cd	7.9 ± 0.4 c	1.78 ± 0.17 bc	0.44 ± 0.04 e	1.11 ± 0.12 de	1.04 ± 0.28 bcd	0.15 ± 0.05 bcd	11.72 ± 3.04 a	89.6 ± 15.5 a
	50	3.47 ± 0.21 d	7.6 ± 0.9 c	1.01 ± 0.12 e	0.11 ± 0.01 fg	0.63 ± 0.03 f	0.57 ± 0.16 ef	0.07 ± 0.02 cd	5.23 ± 1.06 cd	83.2 ± 13.1 a
	30	3.41 ± 0.29 d	11.2 ± 1.1 c	1.14 ± 0.12 de	0.01 ± 0.01 g	0.99 ± 0.12 e	0.38 ± 0.05 f	0.04 ± 0.00 d	1.92 ± 0.37 d	43.5 ± 4.9 bcd
Fo	70	8.16 ± 1.04 bc	9.6 ± 0.5 c	3.07 ± 0.35 a	1.05 ± 0.08 c	1.45 ± 0.13 abc	1.41 ± 0.01 b	0.26 ± 0.01 b	11.98 ± 1.26 a	46.6 ± 4.6 bcd
	50	13.56 ± 1.59 a	9.4 ± 0.6 c	3.27 ± 0.20 a	0.19 ± 0.01 f	1.19 ± 0.09 cde	0.74 ± 0.03 cdef	0.10 ± 0.01 cd	7.73 ± 0.39 bc	77.5 ± 2.7 ab
	30	10.82 ± 2.32 ab	8.0 ± 0.2 c	2.32 ± 0.21 b	0.10 ± 0.00 fg	1.03 ± 0.08 de	0.57 ± 0.01 ef	0.06 ± 0.00 d	4.19 ± 1.19 cd	64.8 ± 15.3 abc
Pr	70	5.00 ± 1.85 cd	10.3 ± 1.3 c	2.33 ± 0.29 b	0.71 ± 0.07 d	1.70 ± 0.10 a	1.12 ± 0.24 bc	0.18 ± 0.06 bc	7.32 ± 1.07 bc	58.8 ± 30.1 abc
	50	4.20 ± 0.89 d	8.6 ± 1.1 c	1.79 ± 0.10 bc	0.14 ± 0.03 fg	1.13 ± 0.12 de	0.82 ± 0.10 cde	0.12 ± 0.02 cd	4.63 ± 0.34 cd	41.7 ± 6.3 bcd
	30	4.16 ± 1.70 d	9.6 ± 1.002 c	1.67 ± 0.12 cd	0.20 ± 0.00 f	1.14 ± 0.07 cde	0.66 ± 0.07 def	0.08 ± 0.01 cd	1.73 ± 0.13 d	21.9 ± 1.4 d
Tm	70	6.38 ± 0.55 cd	38.6 ± 4.1 a	2.04 ± 0.18 bc	1.53 ± 0.08 a	1.65 ± 0.07 a	1.81 ± 0.10 a	0.48 ± 0.10 a	9.27 ± 1.23 ab	19.9 ± 2.1 d
	50	5.13 ± 0.82 cd	28.1 ± 1.6 b	1.94 ± 0.12 bc	1.31 ± 0.06 b	1.32 ± 0.17 bcd	1.41 ± 0.11 b	0.25 ± 0.02 b	7.21 ± 2.11 bc	31.3 ± 12.7 cd
	30	4.26 ± 0.18 d	32.4 ± 4.5b	1.59 ± 0.05 cd	0.10 ± 0.00 fg	1.63 ± 0.13 ab	0.90 ± 0.08 cde	0.13 ± 0.01 cd	4.41 ± 0.73 cd	33.7 ± 2.5 cd

**Table 2:** Soil substrate physicochemical properties. Values are means ± SE (n = 3).

pH_H2O_	8.46 ± 0.04
pH_KCl_	7.83 ± 0.01
EC [µS cm^−1^]	75.75 ± 3.27
N_total_ [% d.w]	0.13 ± 0.00
P [mg kg^−1^ d.w]	318.8 ± 4.4
K [ mg kg^−1^ d.w]	1123 ± 55
Ca [ mg kg^−1^ d.w]	58,330 ± 1510
Mg [ mg kg^−1^ d.w]	30,860 ± 840
P_2_O_5 available_ [mg 100 g ^−1^ d.w]	0.15 ± 0.02
K_2_O _available_ [mg 100 g ^−1^ d.w]	13.4 ± 0.8

## Data Availability

Not applicable.

## Supplementary Materials

The following supporting information can be downloaded at https://www.mdpi.com/article/10.3390/plants12102003/s1, Figure S1: Irrigation curve based on sensor measurements between full water capacity and fully dry soil.
