# Supplementary material for: The Effect of Combined Drought and Temperature Stress on the Physiological Status of Calcareous Grassland Species as Potential Candidates for Urban Green Infrastructure
Source: Plants (Basel). 2023 May 16;12(10):2003. doi: 10.3390/plants12102003 (PMC10222070; doi:10.3390/plants12102003)
Supplement: Supplementary file 1 [file plants-12-02003-s001.zip › plants-2359512-supplementary.pdf]

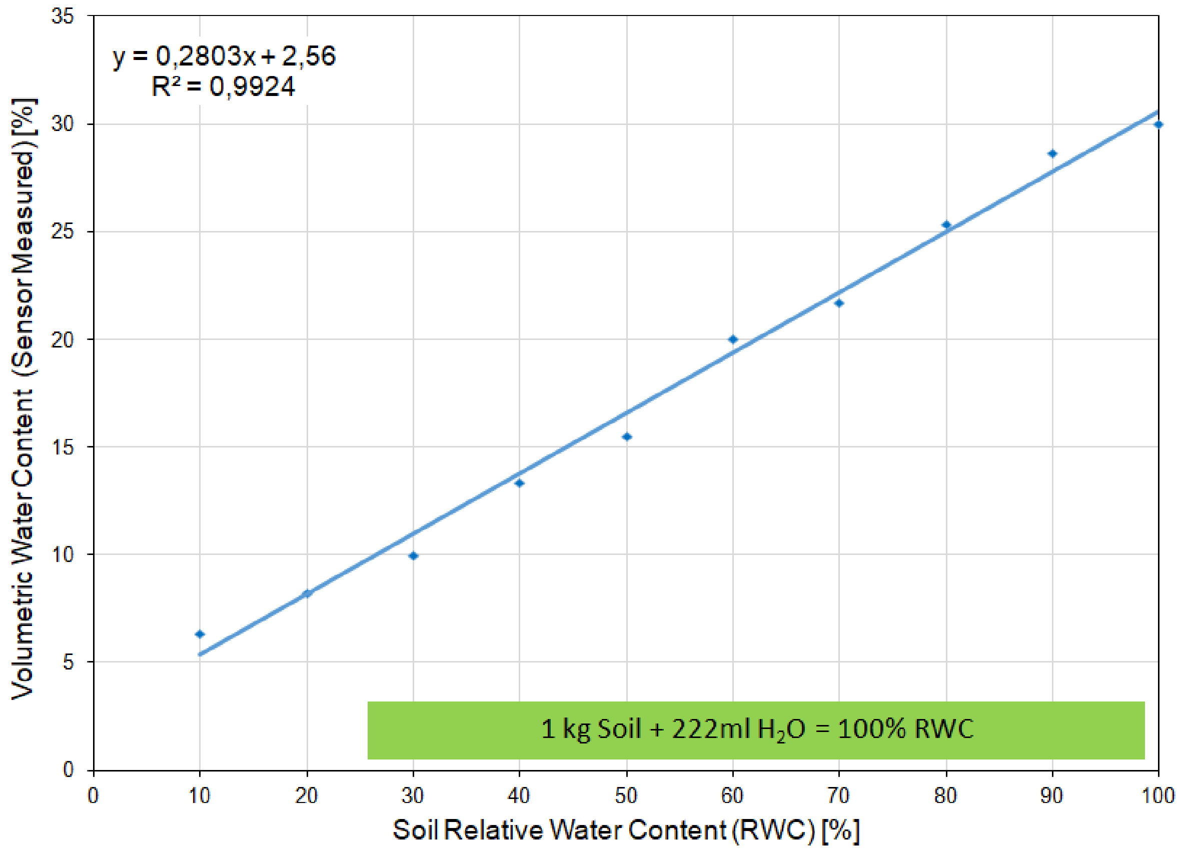


**Figure S1.** Irrigation curve based on sensor measurements between full water capacity and full dry soil. The full water capacity was determined empirically and corresponds to 222 ml $H_2O$ for 1 kg soil. Based on the sensor readings, the pots were supplied with missing % of RWC in the corresponding treatments.
